# *In-vivo* loss of carbapenem resistance by extensively drug-resistant *Klebsiella pneumoniae* during treatment *via* porin expression modification

**DOI:** 10.1038/s41598-017-06503-6

**Published:** 2017-07-27

**Authors:** Suzanne Bialek-Davenet, Noémie Mayer, Julia Vergalli, Marion Duprilot, Sylvain Brisse, Jean-Marie Pagès, Marie-Hélène Nicolas-Chanoine

**Affiliations:** 1 Faculté de Médecine Paris Diderot, Paris, France; 2 Service de Microbiologie, Hôpital Beaujon, AP-HP, Clichy, France; 3 UMR_MD1, Aix-Marseille Univ, IRBA, Marseille, France; 4 INSERM UMR 1137, Université Paris 7, Paris, France; 5 Institut Pasteur, Génomique Evolutive des Microbes, Paris, France

## Abstract

*Klebsiella pneumoniae*, an Enterobacteriaceae that mostly causes hospital-acquired infections, belongs to the recently published WHO’s list of antibiotic-resistant pathogens that pose the greatest threat to human health. Indeed, *K. pneumoniae* is the enterobacterial species most concerned by both resistance to extended-spectrum cephalosporins, due to extended-spectrum β-lactamase (ESBL) production, and resistance to carbapenems, *i.e*. the β-lactams with the broadest activity. Carbapenem resistance is related not only to carbapenemase production, but also the production of ESBL or AmpC and the loss of general porins. Here, we characterized the mechanisms that deprived a urinary ESBL-producing, porin-deficient *K. pneumoniae* isolate, isolated 13 days after the end of a 40-day course of imipenem treatment, of its carbapenem resistance. These mechanisms were observed in two *in-vivo* derivatives of this isolate and consisted of mutations in genes encoding molecules that participate in the downregulation of the synthesis of PhoE, a porin specialized in phosphate transport. We obtained three new derivatives from one of the two original derivatives, following *in-vitro* antibiotic pressure, in which the carbapenem resistance was restored because of mutations in genes encoding molecules that participate in the upregulation of PhoE synthesis. Thus, we uncovered novel mechanisms of carbapenem resistance/susceptibility switching in *K. pneumoniae*.

## Introduction

*Klebsiella pneumoniae* is a Gram-negative pathogen responsible for infections that are mainly hospital-acquired^1, 2^. It was included in the recently published WHO’s list of antibiotic-resistant pathogens that pose the greatest threat to human health (http://www.who.int/mediacentre/news/releases/2017/bacteria-antibiotics-needed/en/), because of its rapid and efficient capacity to develop both multidrug resistance (MDR)^3^ and extensive drug-resistance (XDR)^4^. MDR *K. pneumoniae* isolates consist of those resistant to extended-spectrum cephalosporins, due to extended-spectrum β-lactamase (ESBL) production. XDR isolates consist of those that are also resistant to carbapenems *i.e*. the latest generation of β-lactam molecules that possess the broadest spectrum of activity. Two distinct mechanisms that cause resistance to carbapenems have been identified: acquisition of specific enzymes that hydrolyze carbapenems, called carbapenemases, such as KPC, OXA-48, and NDM^5^, and the combination of ESBL and/or AmpC production and alterations of porin synthesis^6, 7^. The latter is currently the dominant resistance mechanism in various countries^8–10^. However, alterations of porin synthesis have also been described in carbapenemase-producing *K. pneumoniae* isolates^11, 12^, suggesting that the changes in the composition of outer membrane porins which control membrane permeation and the ß-lactam accumulation in the periplasmic space^13^ also plays a key role in the β-lactam resistance displayed by XDR *K. pneumoniae* isolates.

Three consecutive isolates that differed from each other by three carbapenem-susceptibility phenotypes were isolated from a single patient in our hospital during treatment. The first isolate displayed non-carbapenemase-related carbapenem resistance following 40 days of imipenem treatment. The second was susceptible to all carbapenems following 7 days of tigecycline treatment. The third displayed heterogeneous susceptibility to carbapenems following 7-days of tigecycline-colistin treatment. The aim of this study was to identify and characterize the genetic events that occurred in these three urinary isolates responsible for their carbapenem susceptibility. We showed that (i) the three isolates were isogenic strains, each possessing the mechanisms responsible for carbapenem resistance displayed by the first isolate and (ii) the second and third isolates displayed mutations in the *pstSCAB-phoU* genes that control the Pho regulon. We demonstrated that these mutations were involved in the upregulation of the synthesis of the porin PhoE and the complete and partial susceptibility to carbapenems displayed by the second and third isolates, respectively. We subjected the second cefoxitin and carbapenem-susceptible isolate to *in-vitro* cefoxitin and carbapenem selection pressure and showed that resistance to these two antibiotics was restored in the selected mutants due to mutations in the *phoB-phoR* operon, leading to the downregulation of PhoE synthesis. The involvement of *phoE* expression in carbapenem susceptibility had been suggested by Kaczmarek *et al*. following the concomitant isolation of two *K. pneumoniae* strains producing plasmid-mediated AmpC, devoid of general porins, and either susceptible or resistant to carbapenems^14^. However, the authors did not identify the genetic cascades and molecular mechanisms involved in the carbapenem resistance/susceptibility switches.

## Materials and Methods

### Patients and bacterial strains

**Figure 1:**
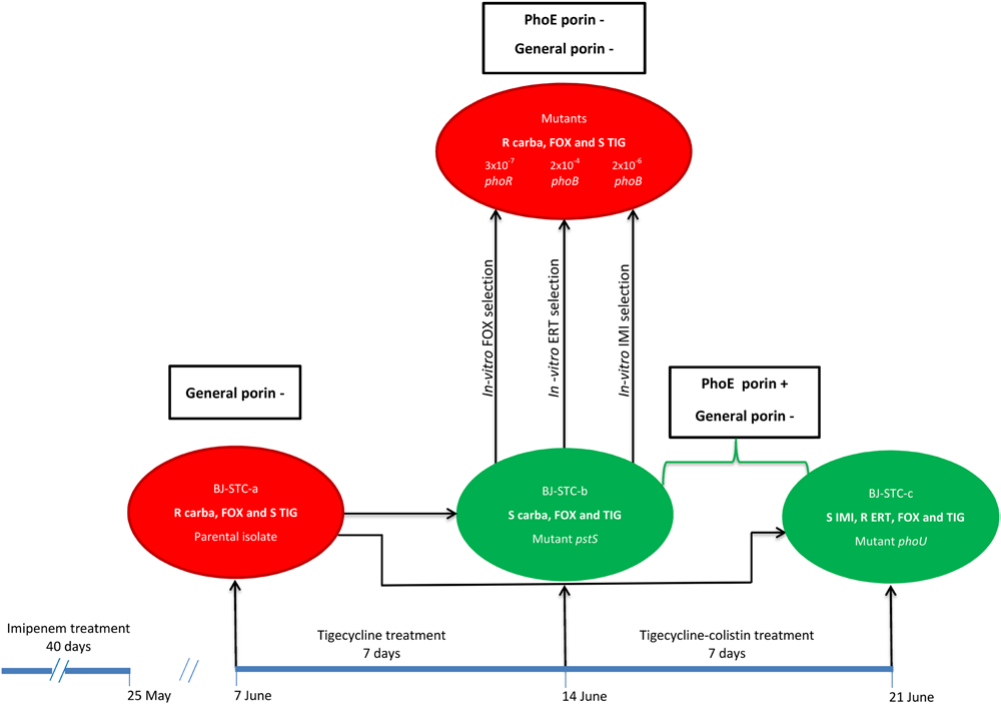
Antibiotic treatment schedule, strain isolation dates, and *in-vitro* strain selection. Phenotypic and genotypic characteristics of the strains are indicated, as well as the selection frequency of the *in-vitro* mutants. Red indicates carbapenem-resistant strains and green indicates strains susceptible to at least one carbapenem. R: resistant; S: susceptible; Carba: carbapenems; FOX: cefoxitin; ERT: ertapenem; IMI: imipenem; TIG: tigecycline.

The patient was a 63-year-old woman of Cambodian origin, who had been living in France for 20 years. She was hospitalized at Beaujon Hospital (AP-HP, Clichy, France) on April 2009 for a fifth recurrent urinary tract infection (UTI). She had been under treatment for disseminated (pulmonary, urinary, and bone) tuberculosis for the previous eight months. Bilateral ureteral damage necessitated the insertion of double J stents in January 2009, which were changed in March 2009. All episodes of recurrent UTI, including that of April 2009, were caused by ESBL-producing *K. pneumoniae* isolates. These infections required iterative treatment with carbapenems. For the fifth episode, the patient received imipenem from April 15 to May 25, 2009, including 10 days after ablation of the double J stents, believed to be responsible for the recurrent UTIs. In June 2009, the patient presented three other recurrences of UTI, demonstrated in the absence of symptoms by a systemic inflammatory response (C-reactive protein = 100 mg/L) and positive urine cultures from systematically collected samples on June 7, 14, and 21 (Fig. 1). The three isolates, called strains BJ-STC-a, BJ-STC-b, and BJ-STC-c, were tested by the agar-diffusion method and interpreted following the recommendations of the French Antibiogram Committee applicable in 2009. They were found to be resistant to extended-spectrum cephalosporins due to ESBL production, demonstrated by the double-disk synergy test^15^, and gentamicin, amikacin, chloramphenicol, cotrimoxazole, and fluoroquinolones (Table 1). They differed from each other by susceptibility to four antibiotics (Table 1): strain BJ-STC-a was resistant to cefoxitin and ertapenem, had intermediate susceptibility to imipenem, and was susceptible to tigecycline, whereas strain BJ-STC-b was susceptible to the four antibiotics, and strain BJ-STC-c was susceptible to imipenem and resistant to cefoxitin, ertapenem, and tigecycline. Following the isolation of strain BJ-STC-a, the patient received tigecycline (Fig. 1). After seven days of this treatment, strain BJ-STC-b, susceptible to tigecycline, was isolated. Therefore, colistin (MIC: 1 mg/l determined by E test) was added to the treatment (Fig. 1), with doses adapted to patient renal function (1 MU in IM injection every two days). Although strain BJ-STC-c was isolated under the combined treatment, this treatment was maintained until July 16, 2009. A urine analysis performed on July 27, 2009 showed only the presence of vaginal flora and the absence of *K. pneumoniae*. Strains BJ-STC-a, BJ-STC-b, and BJ-STC-c were studied with strain ATCC 13883^T^ used as a control.

**Table 1:** Antibiotic susceptibility of the three ESBL-producing *Klebsiella pneumoniae* strains studied.

Strain	Isolation date	Antibiotic susceptibility tested in 2009^a^												
		AMC	FOX	CTX	CAZ	ETP	IMP	GEN	AMI	TET	TGC	CMP	SXT	CIP
BJ-STC-a	6/07/2009	R	R	R	R	R	I	R	R	R	S	R	R	R
BJ-STC-b	6/14/2009	R	S	R	R	S	S	R	R	R	S	R	R	R
BJ-STC-c	6/21/2009	R	R	R	R	R	S	R	I	R	R	R	R	R

^a^Using the agar diffusion method and interpreted according to the 2009 recommendations of the French Antibiogram Committee. AMC: amoxicillin + clavulanic acid; FOX: cefoxitin; CTX: cefotaxime; CAZ: ceftazidime; ETP: ertapenem; IMP: imipenem; GEN: gentamicin; AMI: amikacin; TET: tetracycline; TGC: tigecycline; CMP: chloramphenicol; SXT: cotrimoxazole; CIP: ciprofloxacin. R: resistant, S suceptible, I : intermediate susceptible.

### Genome sequencing and analysis

The complete genomic sequence was determined for strains BJ-STC-a, BJ-STC-b, and BJ-STC-c. Total DNA was extracted using the Qiagen Blood & Cell Culture DNA Mini Kit (Qiagen, Courtaboeuf, France). Libraries were constructed using Nextera technology and sequenced on an Illumina HiSeq-2000 using a 2 × 100 nucleotide (nt) paired-end strategy. All reads were processed to remove low quality or artefactual nucleotides, using sequentially sickle (github.com/najoshi/sickle), AlienTrimmer^16^, and fqDuplicate (ftp.pasteur.fr/pub/gensoft/projects/fqtools). Read pairs were assembled using clc assembler from the CLC Genomics Workbench analysis package (www.clcbio.com/products/clc-genomics-workbench). All contigs of ≥500 nt were reordered and reoriented, using the genomic sequence of strain NTUH-K2044 as reference, with Mauve Contig Mover^17^. The reordered contigs were subsequently imported into the Bacterial Isolate Genome Sequence Database (BIGSdb) platform^18^ and analyzed for the presence of known resistance-associated genes, as well as MLST and cgMLST profiles^19^. Resistance genes were also sought using ResFinder with default parameters (https://cge.cbs.dtu.dk/services/ResFinder)^20^. The putative IS identified in the genome were characterized using ISFinder (www-is.biotoul.fr). Plasmid content was determined using PlasmidFinder (https://cge.cbs.dtu.dk/services/PlasmidFinder) and plasmid type by using pMLST (https://cge.cbs.dtu.dk/services/pMLST)^21^. The reads corresponding to strains BJ-STC-b and BJ-STC-c were mapped on the contigs of strain BJ-STC-a, to search for differences between the three genomes, using the Quality-based Variant Detection tool of CLC Genomics Workbench 7.0.3 with default parameters.

### Mutant selection

The mutants BJ-STC-b-M_FOX_, BJ-STC-b-M_ERT_, and BJ-STC-b-M_IMP_ were obtained from strain BJ- STC-b by *in vitro* selection using cefoxitin, ertapenem, and imipenem, respectively, following the procedure described previously^22^.

### Sequence studies

We used Sanger sequencing, with the primers listed in Table S1 and previously described PCR and sequencing procedures^22^ to (i) confirm the sequences of the *ompK35*, *ompK36*, *pstS*, *phoU, and oqxR* genes, and the *rarA* and *oqxA* inter-region, and (ii) determine the sequences of the *phoB* and *phoR* genes.

### Analysis of gene expression by real-time RT-PCR

The expression of the genes encoding porins (*ompK35*, *ompK36*, and *phoE*), as well as that of genes encoding regulators and molecules involved in the efflux pumps OqxAB (*rarA* and *oqxB*) and AcrAB (*ramA* and *acrB*) was determined by real-time RT-PCR, as described previously^22, 23^, except for the determination of total RNA concentrations, which was performed using the Qubit RNA BR Assay Kit (Thermo Fischer Scientific, Illkirch, France). All primers used are listed in Table S1.

### Cloning of the *oqxR*, *pstS*, *phoU, phoR*, and *phoB* genes and complementation experiments

The *oqxR* gene from strain ATCC 13883^T^ and the *pstS*, *phoU*, *phoR*, and *phoB* genes of strain BJ-STC-a were cloned into the pCR™8/GW/TOPO® vector (encoding resistance to spectinomycin) following the manufacturer’s instructions (Life Technologies SAS, Saint-Aubin, France). The empty plasmid pCR™8/GW/TOPO®, used as a negative control, was generated from pCR™8/GW/TOPO®-*pstS* after digestion with *Eco*RI enzyme (Ozyme, Saint-Quentin-en-Yvelines, France) and ligation using the LigaFast Rapid DNA Ligation System (Promega, Charbonnières-les-Bains, France). After verification of the constructs by sequencing, the recombinant plasmids were electroporated into competent bacterial cells of strains BJ-STC-a for *oqxR*, BJ-STC-b for *pstS*, BJ-STC-c for *phoU*, BJ-STC-b-M_FOX_ for *phoR*, and BJ-STC-b-M_ERT_ and BJ-STC-b-M_IMP_ for *phoB*. Transformants were selected on LB agar plates containing 100 mg/L spectinomycin.

### Antibiotic susceptibility testing

Minimum inhibitory concentrations (MICs) were determined in triplicate by the broth microdilution method according to the guidelines of EUCAST^24^ and interpreted according to the 2016 CASFM-EUCAST recommendations (www.sfm-microbiologie.org). The following antibiotics were tested in addition to ertapenem and imipenem: cefoxitin, temocillin, tigecycline, chloramphenicol, and colistin. These antibiotics were tested because resistance to them can be associated with defined resistance mechanisms (*e.g*. overexpression of efflux pumps for cefoxitin, chloramphenicol, and tigecycline resistance), because they were not tested (temocillin), or were tested using an inappropriate method (E-test for colistin) in 2009 when the three strains were isolated. A difference between MICs was considered to be significant after two dilutions (*i.e*. a 4-fold difference in MIC).

### Immunodetection of outer membrane proteins

To prepare outer membrane protein as previously described^25^, each strain was grown in three different broth media: Mueller Hinton II (MH II), nutrient broth (NB) with low osmotic strength, increasing *ompK35* gene expression, and nutrient broth containing sorbitol (NBS) with high osmotic strength, increasing *ompK36* gene expression. The same amounts of protein of the final preparations were separated on SDS-PAGE gels. Briefly, samples were solubilized in loading buffer at 96 °C to totally denature the various protein complexes and samples (corresponding to an optical density of 0.02 at 600 nm), loaded on SDS**-**PAGE gels (10% polyacrylamide, 0.1% SDS) and run at 160 V for 90 mn. Electrotransfer of the resulting bands to nitrocellulose membranes was carried out in 0.05% SDS. After an initial saturating step in Tris-buffered saline (TBS: 50 mM Tris-HCl, 150 mM NaCl, pH 8) containing 10% skimmed milk powder overnight at 4 °C, nitrocellulose membranes were incubated in TBS containing skimmed milk powder and 0.2% Triton X-100 for 2 h at room temperature in the presence of polyclonal antibodies directed against OmpA, OmpC, or OmpF proteins. Our previous studies demonstrated strong cross-immunoreactivity between *E. coli* and *K. pneumoniae* porins^26^. Polyclonal antibodies directed against *E. coli* OmpC and OmpF denatured porins have been previously used for the efficient detection of *K. pneumoniae* porins in various clinical and laboratory strains^26, 27^. The detection of resulting antigen-antibody complexes was performed with horseradish peroxidase secondary antibody conjugated goat anti-rabbit IgG antibodies^28^ and revelation was performed using a Chemidoc XRS+ (BioRad, Marnes La Coquette, France).

### Maximal growth rate

For comparative growth assays, strain ATCC 13883^T^ and the various BJ-STC strains were grown overnight (O/N) at 37 °C in Luria-Bertani (LB: 10 g tryptone, 5 g yeast extract, 10 g/L NaCl) in flasks with constant stirring at 280 rpm. Then, O/N cultures were prediluted in LB to obtain an OD_600_ = 0.5 and the strains inoculated into two different wells each at a dilution of 1/10,000 in a Costar® 96 flat-bottomed well plate. The plates were incubated at 37 °C with stirring and growth was recorded by an Infinite 200 Tecan®, which measured the OD_600_ in each well every 5 min, for 24 h. Growth assays were repeated three times. The maximum growth rate (MGR) was calculated from the resulting growth curves. The OD_600_ values in nm were collected and Log-transformed. Curves were calculated from a smoothed spline function. The MGR was defined as the time point at which the maximum value of the derivative of the smoothed function was observed. The MGRs of strains BJ-STC-b, BJ-STC-c, and ATCC 13883^T^ were compared to that of strain BJ-STC-a, allowing determination of the relative growth rate (RGR). Tukey’s test was used for intergroup comparisons and R software for statistical analyses. P values < 0.05 were considered to be statistically significant.

### Nucleotide sequence accession number

The annotated genomic sequences of strains BJ-STC-a, BJ-STC-b, and BJ-STC-c were submitted to the ENA public sequence repository and are available under project accession number PRJEB14454.

## Results

### Genome analysis and clonal relatedness of the BJ-STC-a, BJ-STC-b, and BJ-STC-c strains

The genome sequences of strains BJ-STC-a, BJ-STC-b, and BJ-STC-c were assembled into 103, 134, and 106 contigs, respectively. MLST analysis performed on whole genome sequence data showed that the three strains belonged to sequence type (ST) 340. Pairwise gene-by-gene comparisons following the cgMLST scheme revealed no differences between strains BJ-STC-a, BJ-STC-b, and BJ-STC-c for the 694 cgMLST genes except for one (the *folE* gene). The *folE* gene was incomplete in the genome assembly of strain BJ-STC-c and therefore no allele was called. Thus, the three strains were isogenic.

### Phenotype and genotype of antibiotic resistance

Strains BJ-STC-a, BJ-STC-b, and BJ-STC-c displayed a similar phenotype of resistance to various antibiotic families (Tables 1 and 2), including extended-spectrum cephalosporins, temocillin, aminoglycosides (gentamicin and amikacin), tetracycline, chloramphenicol, sulphonamide, trimethoprim, and fluoroquinolones. The three strains were susceptible to colistin. Finally, they differed from each other with respect to resistance to carbapenems and cefoxitin (ertapenem and cefoxitin resistance in strains BJ-STC-a and BJ-STC-c and susceptibility to these antimicrobial agents in strain BJ-STC-b; non-susceptibility to imipenem in strain BJ-STC-a and susceptibility in strains BJ-STC-b and BJ-STC-c). They also differed with respect to tigecycline susceptibility, as we observed resistance only in strain BJ-STC-c. Whole genome analysis using ResFinder and BIGSdb revealed the following resistance encoding genes in the three strains: *bla*_SHV-11_, *bla*_TEM-1_, *bla*_OXA-1_, *bla*_CTX-M-15_ (β-lactam resistance), *aph(3*″*)-Ib* and *aph(6)-Id* (streptomycin resistance), *aac(3)-IId* (gentamicin and tobramycin resistance), *aac(6*′*)Ib-cr* (amikacin and fluoroquinolone resistance), *tet(D)* (tetracycline resistance), a truncated chloramphenicol acetyl-transferase-encoding *catB3* gene, *sul2* (sulphonamide resistance), and *dfrA14* (trimethoprim resistance). Mutations in the quinolone resistance determining region (QRDR) were present in genes *gyrA* (S83I) and *parC* (S80I) for the three strains. We detected no carbapenemase-encoding genes in the whole genome sequences of the three strains. We also did not detect the mutation leading to the Val57Leu amino acid substitution in the S10–30S ribosomal protein, considered to be involved in tygecycline resistance, in strain BJ-STC-c, which displayed resistance to tygecycline^29^.

**Table 2:** Antibiotic susceptibility profiles and relative transcription levels of the *ompK35, ompK36, phoE, oqxB, rarA, acrB*, and *ramA* genes for the studied *Klebsiella pneumoniae* strains.

Strain	Gene expression (fold change)							MIC (mg/L)							
	*ompK35*	*ompK36*	*phoE*	*oqxB*	*rarA*	*acrB*	*ramA*	ETP	IMP	FOX	TEM	CMP	TGC	COL	SPC
ATCC 13883^T^	1	1	1	1	1	1	1	≤0.03	0.5	4	4	4	0.5	1	16
BJ-STC-a	0.10 ± 0.01	0.25 ± 0.04	0.89 ± 0.03	68 ± 45	623 ± 460	0.48 ± 0.21	0.34 ± 0.06	128	8	64	128	64	0.5	1	64
BJ-STC-a T_*oqxR*wt_	0.04 ± 0.03	0.19 ± 0.08	0.71	10 ± 7	84 ± 71	0.46 ± 0.16	0.24 ± 0.04	128	8	64	128	32	0.5	1	>1024
BJ-STC-b	0.08 ± 0.04	0.26 ± 0.08	31 ± 13	72 ± 53	641 ± 475	0.70 ± 0.26	0.39 ± 0.25	0.25	1	8	128	64	0.5	1	32
BJ-STC-b T_*pstS*wt_	0.18 ± 0.03	0.75 ± 0.06	0.95 ± 0.55	53 ± 2	1036 ± 100	0.56 ± 0.12	0.79 ± 0.35	>128	8	64	128	64	0.5	2	>1024
BJ-STC-c	0.07 ± 0.01	0.30 ± 0.07	4.70 ± 0.57	60 ± 15	365 ± 88	1.46 ± 0.21	14 ± 3	32	2	128	128	128	4	1	64
BJ-STC-c T_*phoU*wt_	0.12 ± 0.02	0.50 ± 0.08	0.72 ± 0.31	64 ± 17	712 ± 132	1.33 ± 0.26	18 ± 4	128	8	128	128	128	2	2	>1024
BJ-STC-b-M_FOX_	0.05 ± 0.01	0.34 ± 0.02	0.38 ± 0.13	19 ± 6	532 ± 473	0.43 ± 0.01	0.35 ± 0.07	128	8	64	256	64	0.5	2	32
BJ-STC-b-M_FOX_ T_*phoR*wt_	0.18 ± 0.01	0.66 ± 0.01	22 ± 15	56 ± 22	1081 ± 243	0.48 ± 0.03	0.92 ± 0.31	0.5	1	16	128	64	0.5	2	>1024
BJ-STC-b-M_ETP_	0.05 ± 0.04	0.18 ± 0.04	0.40 ± 0.28	88 ± 67	769 ± 630	0.39 ± 0.08	0.28 ± 0.03	128	8	64	128	64	0.5	1	64
BJ-STC-b-M_ETP_ T_*phoB*wt_	0.05 ± 0.04	0.15 ± 0.03	2.90 ± 0.44	49 ± 28	418 ± 243	0.57 ± 0.12	0.22 ± 0.01	0.5	0.5	32	128	64	0.5	1	>1024
BJ-STC-b-M_IMP_	0.05 ± 0.04	0.15 ± 0.04	0.42 ± 0.24	67 ± 51	503 ± 395	0.36 ± 0.09	0.31 ± 0.16	128	8	64	128	64	0.5	1	64
BJ-STC-b-M_IMP_ T_*phoB*wt_	0.06 ± 0.05	0.20 ± 0.06	4.78 ± 0.09	59 ± 41	459 ± 367	0.60 ± 0.19	0.20 ± 0.04	0.25	0.5	32	128	64	0.5	1	>1024

wt: wild type, ETP: ertapenem; IMP: imipenem; FOX: cefoxitin; TEM: temocillin; TGC: tigecycline; CMP: chloramphenicol; COL: colistin; SPC: spectinomycin.

### Plasmid content

Genome sequence analysis showed three plasmid replicons: IncFIIk, IncFIA, and IncR in the three strains of BJ-STC. The pMLST profile displayed by the IncF replicons was K1:A13:B-.

### OmpF and OmpC porin profiles (RT-PCR, sequence, immunodetection)

**Figure 2:**
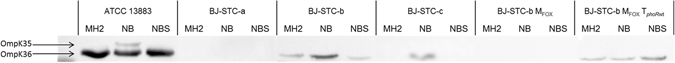
Immunodetection of OmpK35 and OmpK36 synthesis. This detection was performed using polyclonal antibodies directed against denatured OmpC (OmpK 36 in *Klebsiella pneumoniae*) porin. Anti-OmpC antibodies are also able to detect denatured OmpF (OmpK35 in *K. pneumoniae*) porin due to cross-recognition. The tested strains, BJ-STC-a, BJ-STC-b, BJ-STC-c, BJ-STC-b M_FOX_, and BJ-STC-b M_FOX_ T_*phoR*wt_, were grown in Mueller Hinton II (MH2), nutrient broth (NB), or nutrient broth containing sorbitol (NBS) media. Only the relevant part of the blots is shown.

Carbapenem resistance can be due to porin alteration and ESBL production. Thus, we assessed the expression level of the general porins. The three BJ-STC strains expressed the *ompK36* and *ompK35* genes at approximately the same level, which was much lower than in strain ATCC 13883^T^ (Table 2). Comparison of genomic sequence data with the genome of reference strain MGH78578 (GenBank: CP000647.1) showed that the *ompK36* gene was similarly mutated in strains BJ-STC-a, BJ-STC-b, and BJ-STC-c. An insertion of a thymidine at position 4 of this gene resulted in a stop codon directly after the start codon, preventing the synthesis of a functional OmpK36 protein. The *ompK35* gene presented a deletion of one of the three G nucleotides located at position 871 (out of 927 bps) in the three strains, leading to a shift in the reading frame. Moreover, an IS of the IS*1* family was located seven nucleotides upstream from the start codon of the *ompk35* gene in the three strains. The immunodetection experiments (Fig. 2 and Figure S1) showed that neither OmpK35 nor OmpK36 were produced by strain BJ-STC-a, regardless of the broth used for bacterial growth. We detected a band which migrated faster than that of wildtype OmpK36 in strains BJ-STC-b and BJ-STC-c, for which the synthesis of OmpK35 and OmpK36 was also altered. It was detected in strain BJ-STC-b when grown in MH II and NB broths (Fig. 2 and Figure S1) and in strain BJ-STC-c when grown in NB broth (Fig. 2). These results show the expression of another antigenically-related porin recognized by anti-porin antibodies in strains BJ-STC-b and BJ-STC-c.

### Genome comparison and *phoE* expression

**Figure 3:**
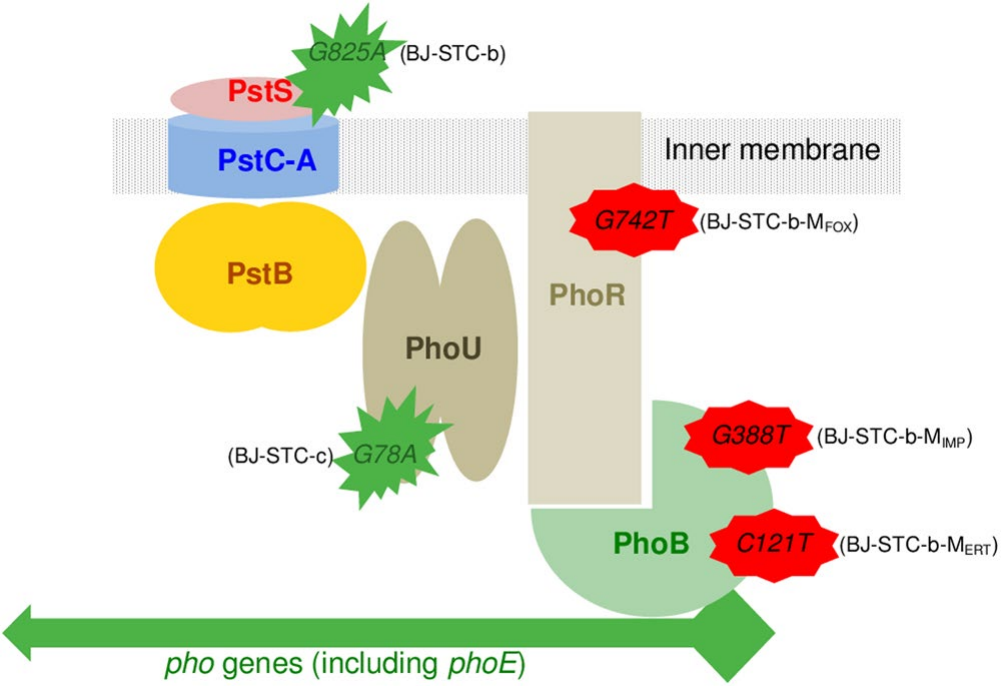
Pho regulon and location of the mutations detected in the various strains. The various molecules encoded by the *pstSCAB* and *phoU-B genes* involved in the control of Pho regulon expression are indicated (adapted from reference Gardner 43). The green and red stars represent the mutations identified that activate or repress the *phoE* expression, respectively.

The three genomic sequences were compared by performing read mapping analysis to define the genetic basis of imipenem-susceptibility in strains BJ-STC-b and BJ-STC-c, and imipenem-resistance in strain BJ-STC-a. We detected potentially meaningful differences in the *pstS* and *phoU* genes, which regulate the expression of genes encoding phosphate transporters^30, 31^. We identified the mutation G825A (Fig. 3), resulting in the replacement of Trp at position 275 by a stop codon, in the *pstS* gene of strain BJ-STC-b, whereas the *phoU* gene of strain BJ-STC-c harbored mutation G78A (Fig. 3), resulting in a Met26Ile substitution. We subsequently verified the two mutations identified by genomic sequence analysis by Sanger sequencing. We measured the level of *phoE* transcripts, as the *pstS* and *phoU* genes regulate the expression of the *phoE* gene. The expression of *phoE* in strain BJ- STC-a was comparable to that of the reference strain ATCC 13883^T^, whereas it was 36-fold and almost 5-fold overexpressed in BJ-STC-b and BJ-STC-c, respectively (Table 2 and Fig. 2).

### *In vitro* reproduction of the carbapenem-resistant phenotype in derivatives of strain BJ-STC-b

Growth of the cefoxitin- and carbapenem-susceptible strain BJ-STC-b in the presence of cefoxitin resulted in the selection (3 × 10^−7^) of a mutant, BJ-STC-b-M_FOX_, with a β-lactam susceptibility pattern identical to that of strain BJ-STC-a: an eight-fold increase in the MIC of cefoxitin and imipenem and a 512-fold increase in the MIC of ertapenem (Table 2). The synthesis of PhoE was downregulated in this mutant, shown by RT-PCR and immunodetection (Table 2, Fig. 2). Sanger sequencing of the *phoB* and *phoR* genes, which are involved in the regulation of *phoE* expression, revealed a G742T mutation (Fig. 3) responsible for a premature stop codon (Glu248Stop) in the *phoR* gene of strain BJ-STC-b-M_FOX_. We used ertapenem and imipenem as selectors to obtain derivatives from strain BJ-STC-b, called BJ-STC-b-M_ERT_ (2 × 10^−4^) and BJ-STC-b-M_IMP_ (4 × 10^−6^), respectively, that exhibit the same β-lactam susceptibility profile as strain BJ-STC-b-M_FOX_ (Table 2). In these mutants, the expression of the *phoE* gene was also downregulated (Table 2) and we identified mutations within the regulator *phoB* gene: stop codons replaced the amino acid Gln at position 41 (C121T) in strain BJ-STC-b-M_ERT_, and amino acid Glu at position 130 (G388T)) in strain BJ-STC-b-M_IMP_ (Fig. 3).

### Complementation experiments

We assessed the role of the mutations located in the *pstS, phoU, phoR*, and *phoB* genes in the modification of *phoE* gene expression by cloning the corresponding genes amplified from the parental BJ-STC-a strain and using them to transform strains BJ-STC-b, BJ-STC-c, and the derivatives of BJ-STC-b. Complementation had an impact on both *phoE* expression and susceptibility to three of the seven antibiotics tested, namely ertapenem, imipenem, and cefoxitin (Table 2). In strains BJ-STC-b T_*pstS*wt_ and BJ-STC-c T_*phoU*wt_, *phoE* expression was downregulated and the MICs of ertapenem, imipenem, and cefoxitin increased to reach the values observed in strain BJ-STC-a. Conversely, *phoE* expression was upregulated and the MICs of cefoxitin, imipenem, and ertapenem decreased in strains BJ-STC-b-M_FOX_ T_*phoR*wt_, BJ-STC-b-M_ETP_ T_*phoB*wt_, and BJ-STC-b-M_IMP_ T_*phoB*wt_. However, the increase of *phoE* expression was less marked in strains BJ-STC-b-M_ETP_ T_*phoB*wt_ and BJ-STC-b-M_IMP_ T_*phoB*wt_ than in strain BJ-STC-b-M_FOX_ T_*phoR*wt_ (Table 2).

### OqxAB and AcrAB efflux pump expression

The resistance to chloramphenicol observed in the three strains, in the absence of an intact *cat* gene, and the marked increase in the tigecycline MIC in strain BJ-STC-c, in the absence of modification of the S10–30S ribosomal protein, led us to analyze the genes encoding the OqxAB and AcrAB efflux pumps, for which overexpression is known to contribute to the resistance to these antibiotics^23^. Transcript levels of the *oqxB* gene and that of the *rarA* gene, encoding a positive regulator of the expression of the *oqxB* gene, were higher in strains BJ-STC-a, BJ-STC-b, and BJ-STC-c than in the reference strain ATCC 13883^T^ (Table 2). The Val130Ala substitution in the *oqxR* gene, *i.e*. the repressor of both *oqxAB* and *rarA* gene expression, was present in all three strains. However, in strain BJ-STC-a T_*oqxR*wt_ *, i.e*. strain BJ-STC-a complemented with the wild type gene *oqxR* of strain ATCC 13883^T^, the expression of the *oqxB* and *rarA* genes was only partially downregulated. This suggests that an additional mechanism, other than the Val130Ala substitution, was involved in both the overexpression of the *oqxB* and *rarA* genes in strain BJ-STC-a and the partial downregulation of these two genes in strain BJ-STC-a T_*oqxR*wt_ (Table 2). We identified another genetic event in the three strains, namely the insertion of an additional G in the seven-G motif located between the *rarA* and *oqxR* genes at positions −123 to −129 upstream of the start codon of the *rarA* gene, corresponding to the DNA*-*binding domain of the winged helix-turn-helix of OqxR^32^. As a result, the MIC of chloramphenicol, a substrate of the OqxAB efflux pump, was slightly lower in strain BJ-STC-a T_*oqxR*wt_ than in strain BJ-STC-a (Table 2).

Although each BJ-STC strain harbored an additional lysine (related to a T580A mutation) at the very end of RamR, the repressor of *acrB* and *ramA* expression, expression of these two genes was appreciably higher in strain BJ-STC-c only (Table 2). This overexpression was due to the insertion of IS*Kpn18*, belonging to the IS*3* family, in the *ramR* gene. Therefore, the MIC of tigecycline, which is a substrate of the AcrB efflux pump and not of the OqxAB efflux pump, was significantly higher in strain BJ-STC-c (4 mg/L) than in strains BJ-STC-a and BJ-STC-b (0.5 mg/L) (Table 2). In contrast, the MIC of chloramphenicol, which is a substrate of both the AcrAB and OqxAB efflux pumps, was not significantly higher in strain BJ-STC-c than in strains BJ-STC-a and BJ-STC-b.

### Relative maximal growth rate (MGR)

**Figure 4:**
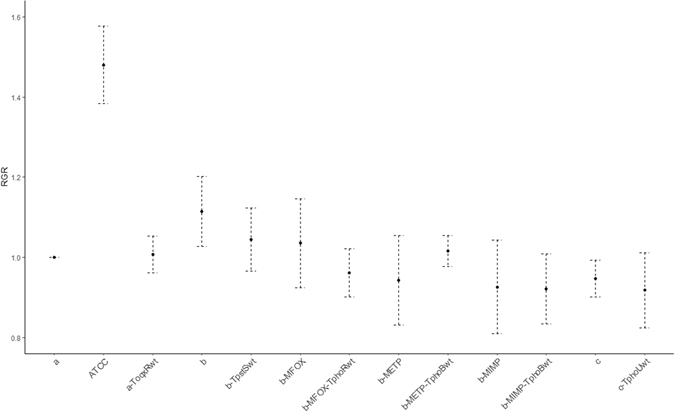
Relative maximal growth rate. The relative growth rate is expressed as the average of the ﻿three growth﻿﻿ determinations ± standard deviation. a: strain BJ-STC-a; a-ToqxRwt: strain BJ-STC-a complemented with the wild type *oqxR* gene; b: strain BJ-STC-b; b-TpstSwt: strain BJ-STC-b complemented with the wild type *pstS* gene; b-MFOX: BJ-STC-b mutant selected with cefoxitin; b-MFOX-TphoRwt: BJ-STC-b mutant selected with cefoxitin and complemented with the wild type *phoR* gene; b-METP: strain BJ-STC-b mutant selected with ertapenem; b-METP-TphoBwt: strain BJ-STC-b mutant selected with ertapenem and complemented with the wild type *phoB* gene; b-MIMP: BJ-STC-b mutant selected with imipenem; b-MIMP-TphoBwt: BJ-STC-b mutant selected with imipenem and complemented with the wild type *phoB* gene; c: strain BJ-STC-c; c-TphoUwt: strain BJ-STC-c complemented with the wild type *phoU* gene.

The MGR of the three BJ-STC strains were significantly lower than that of strain ATCC 13883^T^ (p < 0.001) (Fig. 4). However, the relative MGR of strain BJ-STC-b was non-significantly higher than that of strains BJ-STC-a and BJ-STC-c. The partial decrease in the OqxAB pump activity in strain BJ-STC-a T_*oqxR*wt_, and the repression of the PhoE expression in the complemented strains BJ-STC-b T_*pstS*wt_ and BJ-STC-c T_*phoU*wt_, as well as the BJ-STC-b derivatives (BJ-STC-b-M_FOX_ T_*phoR*wt_, BJ-STC-b-M_ETP_ T_*phoB*wt_ and BJ-STC-b-M_IMP_ T_*phoB*wt_), did not result in relative MGRs significantly different from those displayed by the respective parental strains.

## Discussion

The involvement of combined AmpC overproduction and porin loss in the resistance to imipenem following imipenem treatment has been known since 1991^33^. Since the middle of the 2000s, not only AmpC, but also ESBL, combined with porin loss was found to be responsible for carbapenem resistance^34–36^. This is certainly because of the commercialization and laboratory testing of ertapenem, of which the activity is more strongly affected by synergistic mechanism-mediated carbapenem resistance than that of imipenem^37, 38^. The first urinary *K. pneumoniae* strain (BJ-STC-a) studied herein, which was resistant to carbapenems, resembled those described in various UK hospitals in 2009 in terms of ESBL type produced (CTX-M-15) and genetic events leading to the loss of the general porins OmpK35 and Ompk36 (IS*1* or nucleotide insertion)^37^. However, contrary to the UK strains, strain BJ-STC-a also displayed overexpression of the OqxAB efflux pump, known to expel various antibiotics (*i.e* cefoxitin, chloramphenicol, and fluoroquinolones), generating low level resistance to these antibiotics^23^. This was the sole mechanism responsible for chloramphenicol resistance in strain BJ-STC-a, whereas other mechanisms contributed to its resistance to cefoxitin (porin loss) and fluoroquinolones (target mutations). Strain BJ-STC-a displayed an ST rarely described until recently, ST340^39–41^, which is a member of clonal group 258, to which the worldwide KPC-producing strains belong^42^. The originality of our study is the analysis of two urinary strains sequentially isolated after a seven-day treatment with tigecycline (strain BJ-STC-b) and a seven-day treatment with tigecycline-colistin (strain BJ-STC-c). The study showed that the BJ-STC-a, BJ-STC-b, and BJ-STC-c strains were isogenic, all equipped with the various resistance mechanisms characterized in strain BJ-STC-a, notably those causing resistance to carbapenems. However, the second and the third strains displayed a carbapenem susceptibility profile different from that of the first strain. We demonstrated that the susceptibility of the second strain, BJ-STC-b, to carbapenems, as well as cefoxitin, was caused by a mutated *pstS* gene, leading to constitutive expression of the *phoE* gene. A clinical isolate of *K. pneumoniae* devoid of general porins, producing ESBL, and susceptible to both carbapenems and cefoxitin because of upregulation of PhoE synthesis cannot be phenotypically distinguished from one widely susceptible to carbapenems and cefoxitin. Thus, physicians are likely to use these antibiotics to treat infections due to the former strains. This led us to grow strain BJ-STC-b in the presence of cefoxitin, ertapenem, or imipenem *in vitro*. We obtained a *phoR* mutant when cefoxitin was used whereas two different *phoB* mutants were obtained when ertapenem and imipenem were used. These mutations led to both downregulation of the *phoE* gene and restoration of cefoxitin and carbapenem resistance in the two mutant types because PhoR, an inner-membrane histidine kinase sensor protein, and PhoB, a response regulator that acts as a DNA-binding protein to active or inhibit gene transcription, are the elements of the two-component regulatory system that governs the Pho regulon (Fig. 3)^30, 43^. Our results resemble those obtained by Kaczmarek. *et al*.^14^ in which they grew a carbapenem-susceptible, AmpC-producing, porin-deficient *K. pneumoniae* isolate in the presence of various carbapenems. Indeed, they found that PhoE synthesis was downregulated in the derivatives displaying carbapenem resistance. However, contrary to our study, these authors did not identify the molecular mechanisms responsible for the downregulation of PhoE synthesis. They also did not show why the parental strain was susceptible to carbapenems, whereas it produced an AmpC enzyme and had lost OmpK35 and Ompk36.

In the third strain, BJ-STC-c, which was resistant to both ertapenem and cefoxitin, but susceptible to imipenem, we found constitutive expression of the *phoE* gene, but at a lower level than that observed in strain BJ-STC-b. We found a mutation in the *phoU* gene, of which the product negatively regulates the signaling pathway of inorganic phosphate and modulates its transport through the PstSCAB proteins (Fig. 3)^43^. We used complementation experiments to show that this mutation was linked to *phoE* overexpression, susceptibility to imipenem, and the decrease in the ertapenem MIC relative to the parental BJ-STC-a strain. We postulate that the non-susceptibility to ertapenem found in strain BJ-STC-c, relative to the ertapenem susceptibility found in strain BJ-STC-b, was related to the different amount of ertapenem that accumulated in the periplasm due to the different levels of PhoE upregulation observed in these two strains. The persistence of a high cefoxitin MIC observed for strain BJ-STC-c, but not for strain BJ-STC-b, despite higher *phoE* overexpression, was related to the presence of the overexpression of the AcrAB efflux pump, known to expel cefoxitin, in strain BJ-STC-c. This efflux pump, in contrast to the OqxAB efflux pump, also expels tigecycline, explaining the resistance of strain BJ-STC-c to tigecycline^22, 23^. Finally, we found identical high MIC values of temocillin towards the three BJ-STC strains and the BJ-STC-b derivatives, suggesting that the activity of temocillin is not influenced by the Pho regulon.

The genomic study indicates that the BJ-STC isolates are isogenic strains, suggesting that they were concomitantly present in the urinary tract of the patient, who required double J stents for five months and received repeated imipenem treatments. We postulate that the suspected biofilm on the ureteral device and the constitutive activation of the Pho regulon that, beyond its role in phosphate homeostasis, interacts with the stress response and controls the expression of virulence traits^30, 44^, might have protected the two imipenem-susceptible strains BJ-STC-b and BJ-STC-c from imipenem activity. We also postulate that the different variants, which co-existed as persister bacteria during the antibiotic treatments due to their different metabolisms^45–47^, arose sequentially in the urine, depending on the treatment used. The physiological advantage of the imipenem-susceptible strains, BJ-STC-b and BJ-STC-c, over the imipenem-resistant strain, BJ-STC-a, was not revealed by improved fitness of the two susceptible strains relative to the resistant one. Further studies are required to obtain more insight into the advantages provided by the upregulation of the Pho regulon in ESBL-producing *K. pneumoniae* isolates devoid of OmpK35 and OmpK36. Our *in-vitro* experiments showed that the susceptibility to carbapenems recovered by strains BJ-STC-b and BJ-STC-c is ephemeral. Indeed, variants resistant to carbapenems and cefoxitin due to additional mutations (PhoR, PhoB) located downstream of the mutated regulators (PstS and PhoU) were obtained under carbapenem and cefoxitin pressure, suggesting a risk of *in-vivo* selection of resistant mutants due to downregulation of the Pho regulon.

In conclusion, this is the first study showing that mutations located in the *pstSABC-phoU* operon deprive a XDR *K. pneumoniae* clinical isolate (producing CTX-M-15, devoid of OmpK35 and OmpK36 and overproducing the OqxAB efflux pump) of its resistance to carbapenems and cefoxitin. It also shows that the versatility of the expression of outer membrane porins appears to play a key role in bacterial adaptation and may play an important role in persister cells during antibiotic treatment.

## Electronic supplementary material

Supplementary Information

## References

[1] Podschun R, Ullmann U (1998). *Klebsiella* spp. as nosocomial pathogens: Epidemiology, taxonomy, typing methods, and pathogenicity factors. Clin Microbiol Rev.

[2] Moradigaravand D, Martin V, Sharon P, Parkhill J (2017). Evolution and epidemiology of multidrug-resistant *Klebsiella pneumoniae* in the United Kingdom and Ireland. MBio.

[3] Pendleton J-N, Gorman S-P, Gilmore B-F (2013). Clinical relevance of the ESKAPE pathogens. Expert Rev Anti-Infect Ther.

[4] Du J (2016). Molecular epidemiology of extensively drug-resistant *Klebsiella pneumoniae* outbreak in Wenzhou, Southern China. J Med Microbiol.

[5] Pitout J-D, Nordmann P, Poirel L (2015). Carbapenemase-producing *Klebsiella pneumoniae*, a key pathogen set for global nosocomial dominance. Antimicrob Agents Chemother.

[6] Wozniak A (2012). Porin alterations present in non-carbapenemase-producing *Enterobacteriaceae* with high and intermediate levels of carbapenem resistance in Chile. J Med Microbiol.

[7] Tsai YK (2011). *Klebsiella pneumoniae* outer membrane porins OmpK35 and OmpK36 play roles in both antimicrobial resistance and virulence. Antimicrob Agents Chemother.

[8] Huang, T. D. *et al*. Increasing proportion of carbapenemase-producing Enterobcareriaeae and emergence of MCR-1 producer through a multicentric study among hospital-based and private laboratories in Belgium from September to November 2015. *Eurosurveillance* **22** (2017). 10.2807/1560-7917.ES.2017.22.19.30530 PMC5476986 28537547

[9] Robert J, Pantel A, Merens A, Lavigne JP, Nicolas-Chanoine MH (2014). Incidence rates of carbapenemase-producing *Enterobacteriaceae* clinical isolates in France: a prospective nationwide study in 2011–12. J Antimicrob Chemother.

[10] Wang J-T (2015). Carbapenem-nonsusceptible *Enterobacteriaceae* in Taiwan. PLoS ONE.

[11] Zhang Y (2014). Contribution of β-lactamases and porin proteins OmpK35 and OmpK36 to carbapenem resistance in clinical isolates of KPC-2-producing *Klebsiella pneumoniae*. Antimicrob Agents Chemother.

[12] Garcia-Fernandez A (2012). *Klebsiella pneumoniae* ST258 producing KPC-3 identified in Italy carries novel plasmids and OmpK36/OmpK35 porin variants. Antimicrob Agents Chemother.

[13] Masi M, Réfregiers M, Pos K-M, Pagès J-M (2017). Mechanisms of envelope permeability and antibiotic influx and efflux in Gram-negative bacteria. Nat Microbiol.

[14] Kaczmarek FM, Dib-Hajj F, Shang W, Gootz TD (2006). High-level carbapenem resistance in a *Klebsiella pneumoniae* clinical isolate is due to the combination of *bla*_ACT-1_ β-lactamase production, porin OmpK35/36 insertional inactivation, and down-regulation of the phosphate transport porin PhoE. Antimicrob Agents Chemother.

[15] Jarlier V, Nicolas MH, Fournier G, Philippon A (1988). Extended broad-spectrum β-lactamases conferring transferable resistance to newer β-lactam agents in *Enterobacteriaceae*: hospital prevalence and susceptibility patterns. Rev Infect Dis.

[16] Criscuolo A, Brisse S (2013). AlienTrimmer: a tool to quickly and accurately trim off multiple short contaminant sequences from high-throughput sequencing reads. Genomics.

[17] Rissman AI (2009). Reordering contigs of draft genomes using the Mauve aligner. Bioinformatics.

[18] Jolley KA, Maiden MC (2010). BIGSdb: Scalable analysis of bacterial genome variation at the population level. BMC Bioinformatics.

[19] Bialek-Davenet S (2014). Genomic definition of hypervirulent and multidrug-resistant *Klebsiella pneumoniae* clonal groups. Emerg Infect Dis.

[20] Zankari E (2012). Identification of acquired antimicrobial resistance genes. J Antimicrob Chemother.

[21] Carattoli A (2014). *In silico* detection and typing of plasmids using PlasmidFinder and plasmid multilocus sequence typing. Antimicrob Agents Chemother.

[22] Bialek-Davenet S (2011). *In vitro* selection of *ramR* and *soxR* mutants overexpressing efflux systems by fluoroquinolones as well as cefoxitin in *Klebsiella pneumoniae*. Antimicrob Agents Chemother.

[23] Bialek-Davenet S (2015). Differential contribution of AcrAB and OqxAB efflux pumps to multidrug resistance and virulence in *Klebsiella pneumoniae*. J Antimicrob Chemother.

[24] EUCAST & ESCMID (2003). Determination of minimum inhibitory concentrations (MICs) of antibacterial agents by broth dilution. Clin Microbiol Infect.

[25] Gayet S, Chollet R, Molle G, Pagès JM, Chevalier J (2003). Modification of outer membrane protein profile and evidence suggesting an active drug pump in *Enterobacter aerogenes* clinical strains. Antimicrob Agents Chemother.

[26] Simonet V, Mallea M, Fourel D, Bolla JM, Pages JM (1996). Crucial domains are conserved in *Enterobacteriaceae* porins. FEMS Microbiol Lett.

[27] Hasdemir UO, Chevalier J, Nordmann P, Pages JM (2004). Detection and prevalence of active drug efflux mechanism in various multidrug-resistant *Klebsiella pneumoniae* strains from Turkey. J Clin Microbiol.

[28] Philippe N (2015). *In vivo* evolution of bacterial resistance in two cases of *Enterobacter aerogenes* infections during treatment with Imipenem. PLoS ONE.

[29] Villa L, Feudi C, Fortini D, García-Fernández A, Carattoli A (2014). Genomics of KPC-producing *Klebsiella pneumoniae* sequence type 512 clone highlights the role of RamR and ribosomal S10 protein mutations in conferring tigecycline resistance. Antimicrob Agents Chemother.

[30] Lamarche MG, Wanner BL, Crepin S, Harel J (2008). The phosphate regulon and bacterial virulence: a regulatory network connecting phosphate homeostasis and pathogenesis. FEMS Microbiol. Rev..

[31] Gardner S-G, Johns K-D, Tanner R, McCleary W-R (2014). The PhoU protein from *Escherichia coli* interacts with PhoR, PstB, and metals to form a phosphate-signaling complex at the membrane. Journal of Bacteriology.

[32] Veleba M, Higgins PG, Gonzalez G, Seifert H, Schneidersa T (2012). Characterization of RarA, a novel AraC family multidrug resistance regulator in *Klebsiella pneumoniae*. Antimicrob Agents Chemother.

[33] Lee EH (1991). Association of two resistance mechanisms in a clinical isolate of *Enterobacter cloacae* with high-level resistance to imipenem. Antimicrob Agents Chemother.

[34] Bidet P (2005). *In vivo* transfer of plasmid-encoded ACC-1 AmpC from *Klebsiella pneumoniae* to *Escherichia coli* in an infant and selection of impermeability to imipenem in *K. pneumoniae*. Antimicrob Agents Chemother.

[35] Skurnik D (2010). Development of ertapenem resistance in a patient with mediastinitis caused by *Klebsiella pneumoniae* producing an extended-spectrum β-lactamase. J Med Microbiol.

[36] Leavitt A (2009). Ertapenem resistance among extended-spectrum-β-lactamase-producing Klebsiella pneumonaie isolates. J Clin Microbiol.

[37] Doumith M, Ellington MJ, Livermore DM, Woodford N (2009). Molecular mechanisms disrupting porin expression in ertapenem-resistant *Klebsiella* and *Enterobacter* spp. clinical isolates from the UK. J Antimicrob Chemother.

[38] Jacoby GA, Mills DM, Chow N (2004). Role of β-lactamases and porins in resistance to ertapenem and other β-lactams in *Klebsiella pneumoniae*. Antimicrob Agents Chemother.

[39] Cerdeira L (2016). Draft genome sequence of a CTX-M-15-producing *Klebsiella pneumoniae* sequence type 340 (clonal complex 258) isolate from a food-producing animal. Journal of global antimicrobial resistance.

[40] Horna G, Velasquez J, Fernandez N, Tamariz J, Ruiz J (2017). Characterization of the first KPC-2-producing *Klebsiella pneumoniae* ST340 in Peru. Journal of Global Antimicrobial Resistance.

[41] Martin WM (2015). Coproduction of KPC-2 and QnrB19 in *Klebsiella pneumoniae* ST340 isolate in Brazil. Diagn Microbiol Infect Dis.

[42] Peirano G (2017). The importance of clonal complex 258 and IncFK2-like plasmids among a global collection of *Klebsiella pneumoniae* with *bla*_KPCs_. Antimicrob Agents Chemother.

[43] Gardner SG (2015). Genetic analysis, structural modeling, and direct coupling analysis suggest a mechanism for phosphate signaling in *Escherichia coli*. BMC Microbiol.

[44] Santos-Beneit F (2015). The Pho regulon: a huge regulatory network in bacteria. Front Microbiol.

[45] Brauner A, Fridman O, Gefen O, Balaban N-Q (2016). Distinguishing between resistance, tolerance and persistence to antibiotic treatment. Nat Rev Microbiol.

[46] Van Acker H, Coenye T (2016). The role of efflux and physiological adaptation in biofilm tolerance and resistance. J Biol Chem.

[47] Van den Bergh B (2016). Frequency of antibiotic application drives rapid evolutionary adaptation of *Escherichia coli* persistence. Nat Microbiol.

