# Supplementary material for: In-vivo loss of carbapenem resistance by extensively drug-resistant Klebsiella pneumoniae during treatment via porin expression modification
Source: Sci Rep. 2017 Jul 27;7:6722. doi: 10.1038/s41598-017-06503-6 (PMC5532282; doi:10.1038/s41598-017-06503-6)
Supplement: Supplementary file 1 — Supplementary Information [file 41598_2017_6503_MOESM1_ESM.pdf]

In-vivo loss of carbapenem resistance by extensively drug-resistant *Klebsiella pneumoniae* during treatment *via* porin expression modification

Suzanne Bialek-Davenet, Noémie Mayer, Julia Vergalli, Marion Duprilot, Sylvain Brisse, Jean-Marie Pagès, Marie-Hélène Nicolas-Chanoine

Supplementary table (Table S1). List of primers used in this study

Assay and target region	Primer	Sequence (5'-3')	Reference
**Sequencing**			
ompK35	ompK35F	CAGACACCAAACTCTCATCA	This work
	ompK35R	TCAGGTTAACGGGAGAATAA	
	ompK35sqF	GCGTCGATCAGAAAGCT	
	ompK35sqR	GCTTCGGCTTTGTCGCCA	
ompK36	ompK36F	TTGCAGCACAATGAAATAGC	This work
	ompK36R	CAAGAGTATACCAGCGAGGT	
	ompK36sqF	CGACGTTCTGCCGGAATT	
	ompK36sqR	AGAGTTACGGTAGGTTGC	
Inter *rarA* and *oxqA*	rarA+intF	ACGGACCGTTCACTATGTCG	37
	rarA+intR	GACGAGCAGAAAGGAGAGCA	
**Sequencing and cloning**			
oqxR	oqxRF	TTCCTGACGCCGGTGTTTTA	37
	oqxRR	CTGCGGTGCCAAAAAGAACA	
pstS	pstSF	CGGCGGTAGATGACAAACGA	This work
	pstSR	CCCCGCCATCTTCGTCAAAA	
	pstSsqF	AAAAATGGGATGACGAAGCG	
	pstSsqR	GGCGCGGCGGACCACGGCGA	
phoU	phoUF	CGTGACTCACAACATGCAGC	This work
	phoUR	GCCGCGGGGTAAAACATATC	
phoB	phoBF	CGTCGTGCTTCAAAATACCC	This work
	phoBR	TTCCATGACAGCCGTTCCAG	
phoR	phoRF	TGGCCATGATCGTATGGTGC	This work
	phoRR	GCAGCGCACGCTCATTAAATA	
	phoRsqF	CGGAACTTTTTCGCCAACGT	
	phoRsqR	CTGCTCCTGCATCATCTCCA	
**RT-PCR**			
acrB	acrBF	CGATAACCTGATGTACATGTCC	Bratu et al.
	acrBR	CCGACAACCATCAGGAAGCT	
ramA	ramAF	ATCGTCGAGTGGATTGATGA	32
	ramAR	AGATGCCATTTCGAATACCC	
oqxB	oqxB1806F	GAGCGAGATCGGGATGAATAC	37
	oqxB1890R	CGGCGTGTTGGTGAACTG	
rarA	Kp02968F	TGGATCGACAACCATCTTGA	23
	Kp02968R	AAGGACTGCTGGGAGTCAAA	
ompK35	ompK35RTF	TCCCTGCCCTGCTGGTAG	Bratu et al.
	ompK35RTR	CTGGTGTCGCCATTGGTGG	

ompK36	ompK36RTF	GCGACCAGACCTACATGCGT	Bratu et al.
	ompK36RTR	AGTCGAAAGAGCCCGCGTC	
phoE	phoEF	TGATGGGCTTTGTGGCTTC	Bratu et al.
	phoER	CTTGCTGTCATAGTCGCTG	
rpoB	rpoBF	AAGGCGAATCCAGCTTGTTCAGC	Bratu et al.
	rpoBR	TGACGTTGCATGTTCGCACCCATCA	

Bratu, S., Landman, D., George, A., Salvani, J. & Quale, J. Correlation of the expression of *acrB* and the regulatory genes *marA*, *soxS* and *ramA* with antimicrobial resistance in clinical isolates of *Klebsiella pneumoniae* endemic to New York City. J Antimicrob Chemother 64, 278-283 (2009).

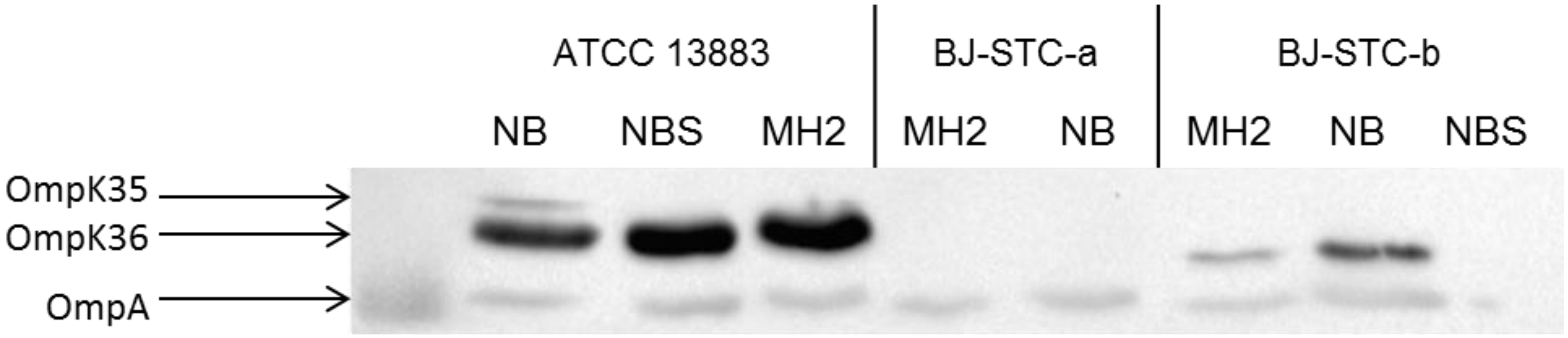

Supplementary figure (Figure S1). Immunodetection of porins and OmpA. After SDS-PAGE separation of denatured samples, the detection was performed with the polyclonal antibodies directed against denatured OmpF (OmpK35 in *Klebsiella pneumoniae*) porin and also against denatured OmpC (OmpK36 in *K. pneumoniae*) porin due to cross-recognition, and OmpA. The tested strains, ATCC13883, BJ-STC-a and BJ-STC-b, were grown in Mueller Hinton II (MH2), nutrient broth (NB) and nutrient broth containing sorbitol (NBS) media. A mix of anti-OmpA and anti-OmpF was used on the same nitrocellulose sheet to detect porins and OmpA.
